# DEclust: A statistical approach for obtaining differential expression profiles of multiple conditions

**DOI:** 10.1371/journal.pone.0188285

**Published:** 2017-11-21

**Authors:** Yoshimasa Aoto, Tsuyoshi Hachiya, Kazuhiro Okumura, Sumitaka Hase, Kengo Sato, Yuichi Wakabayashi, Yasubumi Sakakibara

**Affiliations:** 1 Department of Biosciences and Informatics, Keio University, Yokohama, Kanagawa, Japan; 2 Iwate Tohoku Medical Megabank Organization, Iwate Medical University Disaster Reconstruction Center, Shiwa-gun, Iwate, Japan; 3 Department of Carcinogenesis Research, Division of Experimental Animal Research, Chiba Cancer Center Research Institute, Chiba, Chiba, Japan; Kumamoto University, JAPAN

## Abstract

High-throughput RNA sequencing technology is widely used to comprehensively detect and quantify cellular gene expression. Thus, numerous analytical methods have been proposed for identifying differentially expressed genes (DEGs) between paired samples such as tumor and control specimens, but few studies have reported methods for analyzing differential expression under multiple conditions. We propose a novel method, DEclust, for differential expression analysis among more than two matched samples from distinct tissues or conditions. As compared to conventional clustering methods, DEclust more accurately extracts statistically significant gene clusters from multi-conditional transcriptome data, particularly when replicates of quantitative experiments are available. DEclust can be used for any multi-conditional transcriptome data, as well as for extending any DEG detection tool for paired samples to multiple samples. Accordingly, DEclust can be used for a wide range of applications for transcriptome data analysis. DEclust is freely available at http://www.dna.bio.keio.ac.jp/software/DEclust.

## Introduction

Recently, high-throughput RNA sequencing (RNA-Seq) technology has been used to comprehensively detect and quantify cellular gene expression [1]. Most transcriptomic studies, such as those used in cancer research, are focused on genes whose expression levels differ in a statistically significant manner between experimental conditions, and numerous analytical methods for identifying differentially expressed genes (DEGs) between a pair of matched samples—such as tumor and control specimens—based on RNA-Seq data. edgeR [2], DESeq2 [3], and cuffdiff2 [4] are widely used for comprehensively quantifying gene expression levels and detecting DEGs between a pair of samples or a pair of experimental conditions by using statistical tests; however, to date, few studies have proposed analytical methods for detecting differential expression among multiple conditions.

Cluster analysis according to gene expression patterns is a simple analysis that is performed on multi-conditional transcriptome data for predicting gene functions, searching for biomarkers, and summarizing large datasets; in cluster analysis, sets of objects are grouped in such a manner that objects in the same group (“cluster”) are similar to each other but are different from objects in other groups. For clustering based on gene-expression patterns, Euclidean distance, Pearson’s correlation, and cosine distance are commonly used for measures of distance or similarity between genes [5]. Although the conventional definitions of distance appear to be useful [5], they do not consider any statistical significance based on a dispersion of gene expression: Experimentally quantified gene expression levels possess dispersion due to stochastic biological noise, experimental error, and other factors [4,6], but the conventional definitions of distance consider only the means of expression levels among replicates. Therefore, the existing clustering methods cannot distinguish significant differences in gene expression levels from stochastic biological noise, and, consequently, these methods cannot accurately generate statistically overrepresented patterns of gene expression profiles among multiple conditions from the expression profiles of the genes whose expression levels are significantly changed among the experimental conditions.

To overcome the aforementioned drawbacks associated with analysis methods, we propose here a novel approach for differential expression analysis. Our method, termed DEclust, provides a scoring system for a hierarchical clustering based on differential expression profiles among multiple conditions. We define a collection of statistical test results obtained for all combinatorial pairs of conditions as a pairwise *differential expression test (DET) profile* and assign it to each gene. DEclust searches for a set of statistically overrepresented patterns on DET profiles among multiple conditions by clustering the pairwise DET profiles.

## Results and discussion

### Algorithm

**Fig 1:**
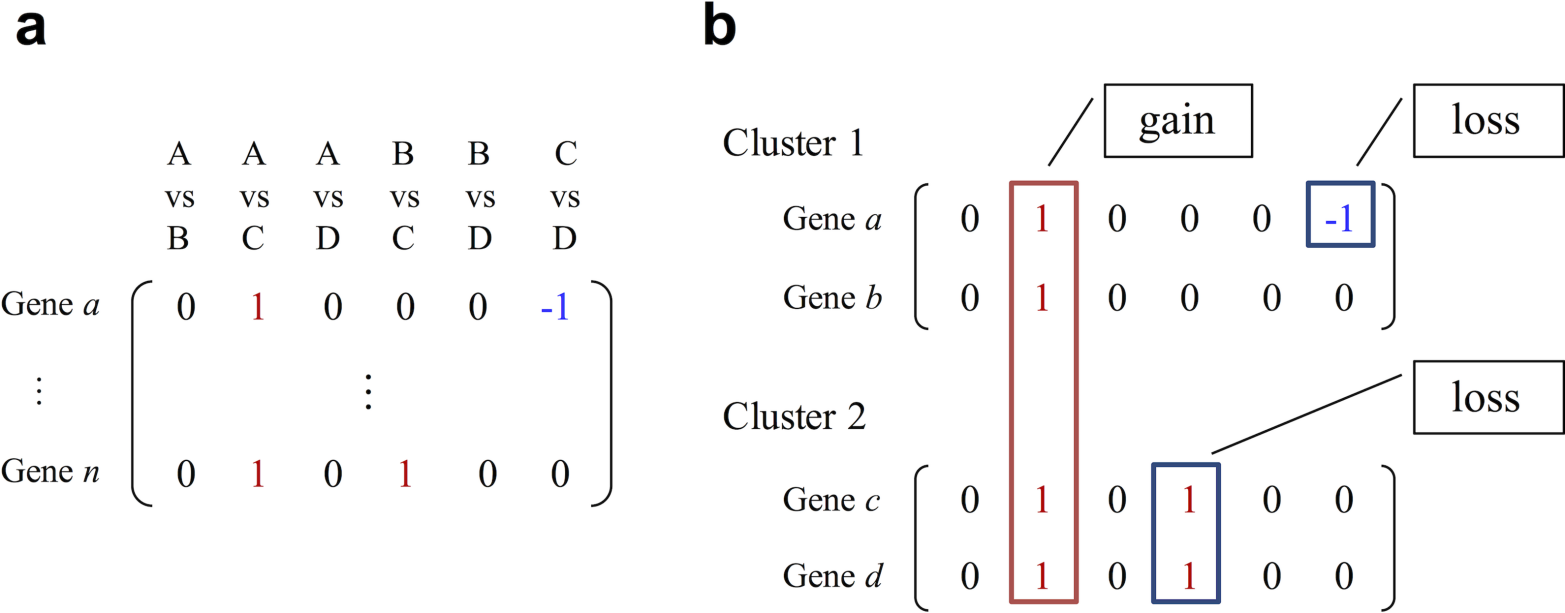
Pairwise DET profile and definition of gain and loss. (a) An example of a pairwise DET profile for genes. All pairwise combinations of four conditions (A, B, C, and D) constitute the six-dimensional pairwise DET profile. (b) An example of the calculation of gains and losses. The pairwise DET profile of Cluster 1 is (0 1 0 0 0 0), and the pairwise DET profile of Cluster 2 is (0 1 0 1 0 0); thus, the pairwise DET profile *v*(*C*_1_∪*C*_2_) is (0 1 0 0 0 0 0). Accordingly, the number of elements that are 1 or -1 in *v*(*C*_1_∪*C*_2_) is 1 (*s* = 1), and that in |*C*_1_∪*C*_2_| is 4; therefore, gain_1,2_ = 4. The total number of elements that are 1 or −1 in *v*{∀*g*∈*C*_*n*_∪*C*_*m*_} is 7 (*t* = 7). Hence, loss_1,2_ = 3 and *D*(*C*_1_, *C*_2_) = 0.857.

For applying a statistical approach to multi-conditional transcriptome data, DEclust adopts a novel distance score between a pair of clusters of genes based on their pairwise DET profiles. The pairwise DET profile is an integer vector constructed from statistical test results for all pairs of conditions. In accordance with common analytical pipelines, RNA-Seq reads are mapped to a reference genome, and an expression level for each gene per condition is estimated. Based on these expression values, a statistical test is applied to all pairs of conditions by using an existing DEG detection tool. The result of the statistical test of differential expression for a pair of conditions is labeled as +1 (upregulated pair), −1 (downregulated pair) or 0 (insignificantly altered pair). Subsequently, these integer values for all pairs of conditions are formed in the vector as a pairwise DET profile. Therefore, the pairwise DET profile features _*N*_*C*_2_ dimensions (*N* is the number of conditions). The expression level of a gene *g* in condition *k* is denoted as *e*_*k*_^*g*^. If the expression level, *e*_*k*_^*g*^, is statistically different from *e*_*l*_^*g*^, then an element of the corresponding dimension in the pairwise DET profile is defined to be 1 (*e*_*k*_^*g*^ < *e*_*l*_^*g*^) or −1 (*e*_*k*_^*g*^ > *e*_*l*_^*g*^); otherwise, it is 0 (Fig 1A). The pairwise DET profile assigned to a gene *g* is defined as follows:

$$v(g)=(d_{1,2}^{g},d_{1,3}^{g},\ldots ,d_{N-1,N}^{g})$$ (1)

$$d_{k,l}^{g}=\left\{ \begin{array}{l} \;+1\;(e_{k}^{g}<e_{l}^{g})\\ \;0\;(no-significance)\\ \;-1\;(e_{k}^{g}>e_{l}^{g}) \end{array}\right.,$$

where *d*_*k*,*l*_^*g*^ represents the statistical test result for the comparison between a pair of certain conditions *k* and *l*.

For inducing statistically overrepresented patterns on DET profiles among multiple conditions, inter-cluster distance is defined to be shorter when a large number of the same statistical test results are shared between the given clusters of genes. DEclust calculates the rate of the statistical test results shared among pairwise DET profiles of genes in the clusters to be merged. First, the definition of the pairwise DET profile is extended to a set (cluster) of genes as follows. An element that corresponds to a statistical test result between a pair of conditions *k* and *l* in the pairwise DET profile for a cluster *C*_*n*_ of genes is defined to be 1 if *d*_*k*,*l*_^*g*^ = 1 for ∀*g*∈*C*_*n*_ and −1 if *d*_*k*,*l*_^*g*^ = −1 for ∀*g*∈*C*_*n*_; otherwise, it is 0. Thus, the pairwise DET profile assigned for a cluster *C*_*n*_ is defined as follows:

$$v(C_{n})=(d_{1,2}^{C_{n}},d_{1,3}^{C_{n}},\ldots ,d_{\left|C|-1,|C\right|}^{C_{n}})$$ (2)

Second, the distance between two clusters *C*_*n*_ and *C*_*m*_ based on the pairwise DET profile is defined as follows:

$$D(C_{n},C_{m})=1-\frac{\left({gain}_{n,m}-{loss}_{n,m}\right)}{\left({gain}_{n,m}+{loss}_{n,m}\right)}.$$ (3)

where gain_*n*,*m*_ is defined as *s*×|*C*_*n*_∪*C*_*m*_|, for *s* denoting the number of elements of +1 or −1 in the pairwise DET profile *v*(*C*_*n*_∪*C*_*m*_) for the union of the clusters *C*_*n*_ and *C*_*m*_; and loss_*n*,*m*_ is defined to be (*t*–gain_*n*,*m*_), for *t* denoting the total number of elements of +1 or −1 in the pairwise DET profiles *v*(*g*) for all genes *g* in the union of the clusters *C*_*n*_ and *C*_*m*_.

Fig 1B shows an example of the gain and loss; the gain and loss are non-negative integer values and if gain_*n*,*m*_ = 0 and loss_*n*,*m*_ = 0, then *D*(*C*_*n*_, *C*_*m*_) = 0. If all pairwise DET profiles of genes in a union of clusters are identical, gain_*n*,*m*_ = *t* and loss_*n*,*m*_ = 0, and thus *D*(*C*_*n*_, *C*_*m*_) = 0. By contrast, if no element has the same value of +1 or −1 among all pairwise DET profiles in a union of clusters, gain_*n*,*m*_ = 0 and loss_*n*,*m*_ = *t*, and therefore *D*(*C*_*n*_, *C*_*m*_) = 2. Thus, *D*(*C*_*n*_, *C*_*m*_) corresponds to an indicator of the shared (or unshared) statistical test result rate in the union of the clusters *C*_*n*_ and C_*m*_.

By using the aforementioned definition of distance, DEclust adopts the agglomerative-hierarchical-type clustering method to identify statistically overrepresented patterns on DET profiles among multiple conditions. DEclust initially merges genes that feature the same statistical test results (i.e., the same pairwise DET profile), and subsequently merges two gene clusters *C*_*n*_ and *C*_*m*_ with minimum distance *D*(*C*_*n*_, *C*_*m*_) so as to conserve the common statistical test results as much as possible. In this manner, DEclust can not only be applied to multi-conditional transcriptome data but also be used to extract statistically significant gene clusters (S1 Text and S1 Algorithm).

### Benchmark study of clustering

For demonstrating the superiority of our method over other methods, we evaluated the accuracy of DEclust and of hierarchical clustering performed with conventional distance measures (hereafter called *existing methods*) by using simulated datasets based on assuming RNA-Seq analysis. In this benchmark study, we used 12 distance measures as the existing methods: the combinations of 4 inter-cluster distances (the group average method, single-linkage method, complete-linkage method, and Ward’s method [7]) and 3 inter-gene distances (Euclidean distance, Pearson’s correlation, and cosine distance). In the text that follows, we denote a DEG as a gene whose expression levels differ in a statistically significant manner between at least one pair of conditions. In the benchmark study, we assumed four artificial conditions, and for evaluation, we established ten pairwise DET profiles as class labels of DEGs (S1 Fig) and one pairwise DET profile for non-DEGs (and thus prepared 11 correct class labels in total). Moreover, 20% of the genes were randomly selected and a class label for DEGs was randomly assigned for each selected gene. Next, we designed the probabilistic distributions as controls for each gene to generate simulated datasets of RNA-Seq read counts. The RNA-Seq read count data of 69 lymphoblastoid cell lines (LCLs) derived from unrelated Nigerian individuals [8] was used as the reference. The read counts for each condition were sampled from the control distribution as many times as the number of replicates, and these were treated as the biological replicates for each condition. Further, the read counts for differentially expressed conditions were relative to a certain fold-change in concordance with the class label (i.e., with the DET profile). According to the read counts, we generated simulated short reads to obtain gene expression profiles for four artificial conditions with a certain number of replicates (S2 Text presents the entire description of simulation datasets). The simulated reads were mapped to a reference genome, and expression levels were estimated for each gene per condition. The statistical tests were applied to all pairs of conditions to detect significant differences in gene expression. We calculated the pairwise DET profile from the results of the statistical tests and assigned it to each gene. Lastly, we implemented DEclust and the existing methods for all of the genes expressed in one or more conditions. The estimation of gene expression levels and the statistical tests were performed using edgeR [2], DESeq [9], DESeq2 [3], and cuffdiff2 [4]. The inputs of DEclust were the pairwise DET profiles and estimated normalized expression levels, and the inputs of the existing methods were the estimated normalized expression levels. In this manner, we performed the simulation thrice for each parameter set. As a result, we generated three simulation datasets in which the genes belonging to the cluster with the same class label and the expression profiles of each gene were different among simulations for each parameter set.

**Fig 2:**
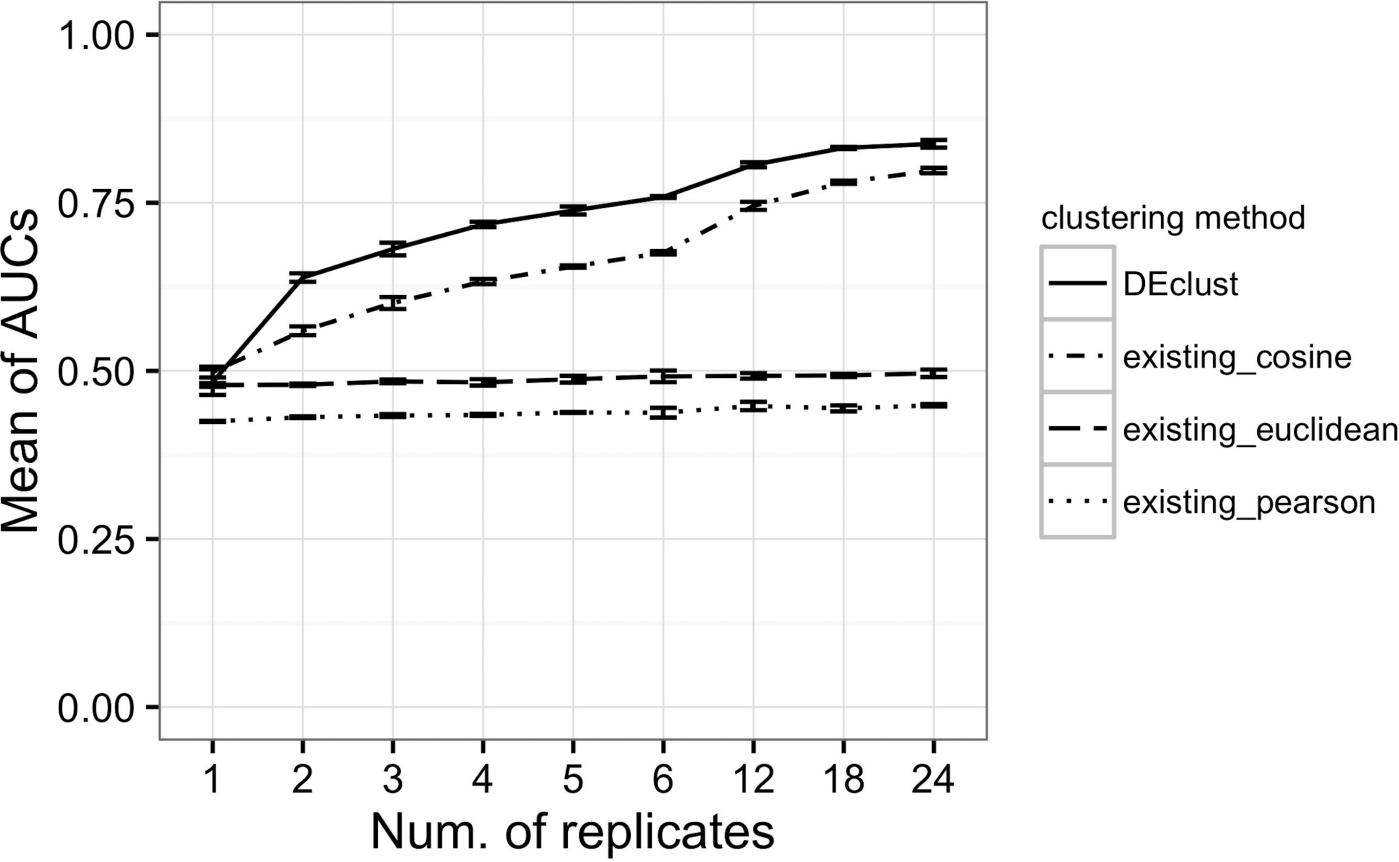
Results of clustering evaluation. *DEclust* is our method and *existing* methods are conventional hierarchical clustering methods. The vertical axis shows the mean AUC values, and the AUCs for each method for each number of replicates are plotted. The error bars are drawn in accordance with the corrected sample standard deviation of three simulations for each parameter set. For existing methods, the group average method was used for the inter-cluster distance measure, and the Euclidean distance, Pearson’s correlation, and cosine distance were used for the inter-gene distance measure. For our method, the evaluation results of DEclust based on the statistical test results of DESeq2 were shown. Also, the group average method with cosine distance was used as the secondary distance measure of DEclust (S1 Text). The complete results are shown in S2–S5 Figs and S1–S4 Tables.

For all pairs of genes featuring identical class labels, we assessed whether they belonged to the same resultant cluster after clustering. We converted the clustering problem into a binary classification problem, and as a measure of the evaluation, we used the area under the curve (AUC) of the receiver operating characteristic (ROC). We calculated the AUCs for each parameter set of inter-gene distances, inter-cluster distances, and number of replicates, and compared the AUCs between DEclust and the existing methods (Fig 2, S2–S5 Figs and S1–S4 Tables). Not only did DEclust show higher accuracy than the existing methods for almost all parameter sets, the AUCs of DEclust were also improved with an increase in the number of replicates. Specifically, DEclust showed superior accuracy in all the datasets containing two or more replicates when DESeq2 was used for statistical tests. Moreover, the AUCs of DEclust were higher than those of the existing methods even when edgeR, DESeq or cuffdiff2 was used with sufficient replicates. Thus, DEclust succeeded in identifying the correct class labels of differentially expressed patterns from multi-conditional transcriptome data more accurately than the existing clustering methods. Conversely, the existing methods showed lower accuracy than DEclust in our benchmark dataset. The existing methods depend solely on the means of the expression levels; therefore, the existing methods could not distinguish significant differences in expression levels from stochastic noise. Consequently, the existing methods could not extract statistically overrepresented and differentially expressed gene clusters (discussed further in S3 and S4 Texts). In either method, the variance of AUCs among the three simulation datasets for each parameter set was not so large.

To summarize this benchmark study, when any biological replicates were available, the highest accuracy was achieved by DEclust with the use of the statistical test results of DESeq2, and when the number of biological replicates was sufficient, DEclust with edgeR was most accurate. Furthermore, if optimal statistical test results are obtained, AUC = 1.00 with our method.

### Benchmark study of DEG detection

Although DEclust uses the results of statistical tests for all combinatorial pairs of conditions, multiDE [10] has been proposed to detect DEGs among multiple conditions. In addition, edgeR and DESeq2 have an extended function, the likelihood ratio test, to detect DEGs among multiple conditions. Therefore, we evaluated the power of DEG detection using multiDE, edgeR, DESeq2, and DEclust. For evaluation of the DEG detection capability of DEclust, we defined a “DEG cluster” and “non-DEG cluster” as follows. If there was at least one element of a pairwise DET profile of a cluster with a value of 1 or −1, the cluster was a DEG cluster; otherwise (that is, all elements were 0), it was a non-DEG cluster. In this manner, DEclust can be applied to the classification problem of whether a gene is a DEG or not. We used the same simulation datasets that were used for the benchmark study to evaluate the performance of DEclust and the conventional clustering methods (the details for the evaluation method are described in the Materials and Methods).

**Fig 3:**
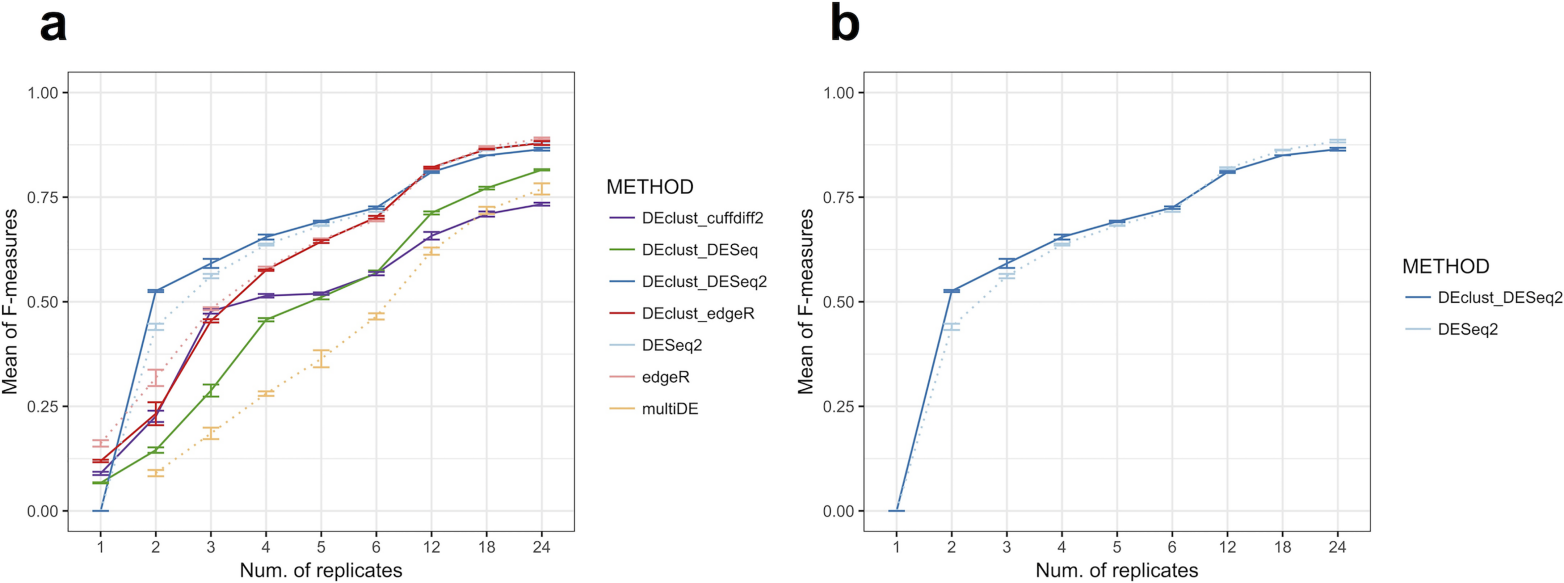
Results of DEG detection evaluation. The F-measures for each method for each number of replicates are plotted. The vertical axis shows the mean F-measures and the error bars are drawn in accordance with the corrected sample standard deviation of three simulations for each parameter set. *DEclust*, our method, uses the statistical test results obtained from edgeR, DESeq, DESeq2, or cuffdiff2, and the evaluation results using each of these tools are separately shown as “DEclust_[DEGs detection tool]”. For the secondary distance of DEclust, the group average method with cosine distance was used. The results for edgeR, DESeq2, multiDE, and DEclust are shown in (a), and the results for only DESeq2 and DEclust with DESeq2 are shown in (b). The complete results are shown in S6 Table.

The performance of edgeR, DESeq2, multiDE, and DEclust was evaluated according to the true-positive rate (TPR), positive predictive value (PPV), accuracy, and F-measure (harmonic mean of TPR and PPV). DEclust uses the pairwise based statistical test results obtained from edgeR, DESeq, DESeq2, or cuffdiff2; the evaluation results using each tool are shown separately (Fig 3, S8 Fig, and S6 Table). Overall, DEclust, based on statistical testing between all pairs of conditions, had higher TPR, and edgeR, DESeq2, and multiDE, based on the statistical testing for multiple conditions, had higher PPV (S8 Fig and S6 Table). Focusing on the F-measure (the harmonic mean of TPR and PPV), DEclust with DESeq2, DEclust with edgeR, sole use of DESeq2, and sole use of edgeR were advantageous relative to the other methods (Fig 3A). Moreover, not only was the DEG detection power of DEclust with DESeq2 and sole use of DESeq2 sufficiently close, but also DEclust with DESeq2 was advantageous when only a few biological replicates were available (Fig 3B).

Although the single statistical test for multiple conditions using edgeR, DESeq2, and multiDE can more accurately identify DEGs, statistical tests for all combinatorial pairs of conditions can identify DEGs with higher sensitivity and determine which pairs of conditions are significantly different. In the present study, the DEG detection ability of DEclust, which was in accordance with the statistical test results for all combinatorial pairs of conditions, was comparable with that of tools that detect DEGs among multiple conditions using a single test. According to the definition of the pairwise DET profile assigned for each cluster (Eq (2)) and the distance between clusters (Eq (3)), the distance between DEG clusters and non-DEG clusters should be 2 (maximum value) because none of the significant statistical test results are shared; therefore, DEclust can accurately cluster the DEGs and non-DEGs if quitting the merge step of hierarchical clustering when the distance between any pair of clusters *n*, *m* satisfies *D*(*C*_*n*_, *C*_*m*_) ≥ 1. Thus, DEclust has high compatibility for multi-conditional transcriptome analysis.

### Application to biological data

To demonstrate the practical usefulness of DEclust, we performed mRNA-Seq analysis on mouse tumor samples and applied DEclust to extract statistically overrepresented and significant gene clusters among multiple stages in the carcinogenesis process. Because carcinogenesis is a multi-step process, time-course analysis is necessary for revealing the carcinogenesis mechanism leading to malignant alteration [11,12]. Based on this perspective, we obtained normal skin, papilloma, carcinoma, and metastatic tumors from each of the same two mice (i.e., two replicates were obtained for each stage), and performed mRNA-Seq on these carcinogenesis samples. We obtained an average of 40.10 M reads by performing mRNA-Seq with an Illumina Genome Analyzer IIx, and we mapped the reads to a mouse reference genome (S7 Table). Subsequently, we identified the genes that were differentially expressed between a pair of the four carcinogenesis stages by using DESeq2. In total, 6,584 DEGs were detected (S8 Table).

**Fig 4:**
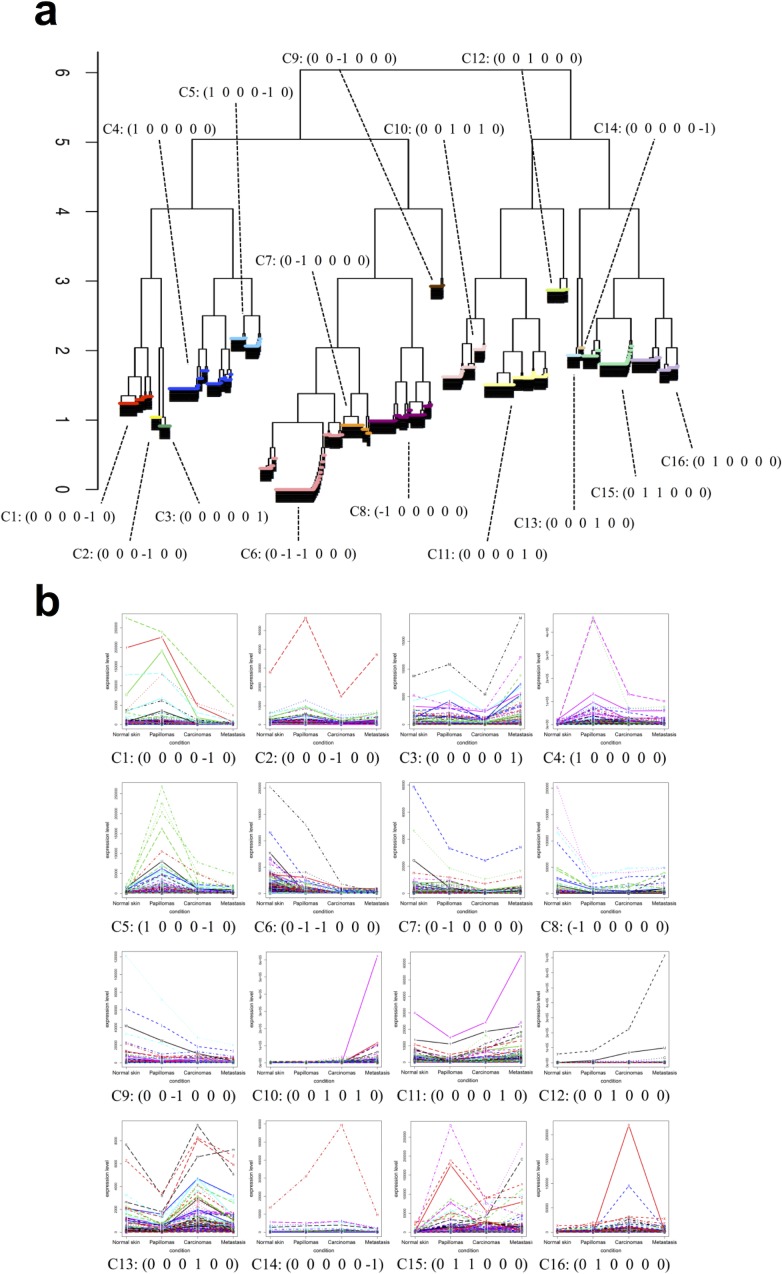
Cancer transcriptome analysis using DEclust. (a) Hierarchical tree of clustering result from DEclust for the transcriptome data. The vertical axis indicates inter-cluster distance (Eq (3)). The DEGs are divided into 16 clusters (which are color-coded). The cluster numbers correspond to the numbers in S10 Table, and each cluster is assigned a pairwise DET profile of six dimensions. (b) Line plots of the expression patterns for each gene in each cluster (the details are shown in S9 Table).

Next, we applied DEclust to the detected 6,584 DEGs between a pair of stages using the results of DESeq2 to extract statistically overrepresented gene clusters among the four carcinogenesis stages. The hierarchical clustering tree of DEGs was calculated, and the DEGs were divided into 16 clusters by quitting the merge step if the distance between any pair of clusters *n*, *m* satisfied *D*(*C*_*n*_, *C*_*m*_) ≥ 1 (Fig 4A). Fig 4B shows the 16 clusters together with the corresponding expression patterns and pairwise DET profiles (the details for each cluster are shown in S9 Table). According to the pairwise DET profiles of the 16 clusters, the genes that belong to one of 16 clusters shared at least one statistical test result of a pair of stages. Thus, DEclust can extract statistically significant gene clusters from multi-conditional transcriptome data. Furthermore, we performed a gene set enrichment analysis (GSEA) for each cluster (the top three hallmarks are shown in S10 Table). The genes of each cluster were annotated with hallmarks such as the *KRAS* signaling pathway, mammalian target of rapamycin (*mTOR*) signaling pathway, and checkpoints of the cell cycle, which are typical cancer-related pathways [12,13].

To compare DEclust and an existing method with the use of the real dataset, we performed hierarchical clustering using the group average method with the cosine distance for the 6,584 DEGs and plotted the hierarchical tree (S9A Fig). The hierarchical tree was cut into 16 clusters to obtain the same number of clusters as in the analysis performed with DEclust. As a result of the assignment of a pairwise DET profile to each cluster, all of the elements in the assigned pairwise DET profiles were zero (S9B Fig). This means that when the existing method was used, the genes showing significant differences and the genes showing non-significant differences in expression levels at the same pair of stages were mixed in the same cluster. Notably, the existing method could not distinguish significant expression differences from noise. Moreover, we performed the GSEA for each cluster generated by the existing method and obtained the top three hallmarks for each cluster (S11 Table). A comparison of the results obtained by DEclust and the existing method revealed that any cluster obtained by DEclust was annotated but several clusters obtained by the existing method could not be annotated because the genes belonging to these clusters were not significantly enriched for biological functions. In addition, interleukin-6/Janus kinase/signal transducer and activator of transcription-3 (*IL6*/*JAK*/*STAT3*) and phosphatidylinositol 3-kinase/protein kinase B/mammalian target of rapamycin (*PI3K*/*AKT*/*mTOR*) signaling pathways were detected only by DEclust. These pathways not only are frequently activated in cancer cells, but are also involved in cancer progression by promoting tumor cell growth, angiogenesis, and metastasis [14,15]. Thus, DEclust can extract biologically significant gene clusters.

## Conclusion

We propose here a novel transcriptome analysis method, DEclust, to search for differentially expressed and statistically overrepresented patterns of gene expression profiles among multiple experimental conditions. The results of the benchmark study show not only that DEclust can extract statistically significant gene clusters from multi-conditional transcriptome data more accurately than conventional clustering methods, but also that the accuracy of DEclust increases if two or more replicates of the quantitative experiments are available. Moreover, the results of the application to biological data (mRNA-Seq analysis of mouse carcinogenesis stages) suggest that DEclust can potentially yield biologically significant gene clusters.

The ready availability of high-throughput sequencing technologies has facilitated large-scale quantitative analyses in all fields of life science. As a result, we expect increased demand for software that is applicable to multi-conditional studies. DEclust can be applied to any multi-conditional transcriptome data, and to the results of any DEG detection tool given an appropriate input format; thus, DEclust can be used for a wide range of applications for transcriptome data analysis into the future. The computational complexity of DEclust is *O*(N^3^), where N is the number of objects (genes). The distances between all pairs of clusters (Eq (3)) must be updated in every iteration of hierarchical clustering. As a reference, when we performed our mouse transcriptome analysis, the computational time of DEclust was approximately ten minutes for 6,584 DEGs with 8 threads of Opteron 1.4 GHz CPUs. The source code of DEclust is freely available at http://www.dna.bio.keio.ac.jp/software/DEclust or http://dx.doi.org/10.17632/wdfb5w7vbb.1. DEclust is implemented using C++ with boost C++ library (http://www.boost.org/) and OpenMP (http://openmp.org/wp/).

## Materials and methods

### Analysis for benchmark study of clustering

For the benchmark study of clustering, simulated reads were mapped to a mouse reference genome (GRCm38-release71) with a gene annotation file by using TopHat2 [16] (version-2.0.8) with Bowtie2 [17] (version-2.1.0). The gene annotations were confined to 14,907 simulated gene annotations (see S2 Text). In this benchmark study, edgeR [2], DESeq [9], DESeq2 [3], and cuffdiff2 [4] were used as statistical tools for detecting significant differential expression between a pair of conditions. At the start of the differential expression analysis, HTSeq [18] (version-0.6.1) was used with the aforementioned confined gene annotation file to count mapped reads for each gene per condition. Subsequently, edgeR (version-3.6.8), DESeq (version-1.16.0), and DESeq2 (version-1.4.5) were used to apply statistical tests for all pairs of conditions to detect significant differential expression. Correspondingly, cuffdiff2 (version-2.2.1) was used with the confined gene annotation file to estimate expression levels and to apply statistical tests. For correcting the multiple testing of differential expression using four tools, we controlled the false discovery rate (FDR). The null hypothesis for multiple conditions was “the expression level of a gene is not different in any of the experimental conditions”; therefore, we estimated the FDR from (total number of genes) × (total number of pairs of conditions) null hypotheses, and controlled it to under 5% by using the Benjamini-Hochberg method [19]. The adjusted p-values were calculated by using R package p.adjust. Consequently, normalized expression values and statistical test results for each gene were obtained from each differential expression analysis tool. Lastly, DEclust and conventional hierarchical clustering algorithms (existing methods) were applied to genes whose normalized expression values were non-zero in at least one condition. For applying DEclust, the statistical test results for each gene were discretized and vectorized to pairwise DET profiles. The group average method, single-linkage method, complete-linkage method, and Ward’s method [7] were used for inter-cluster distance measures, and the Euclidean distance, Pearson’s correlation, and cosine distance were used for inter-gene distance (or similarity) measures for the existing method and for DEclust as secondary distance measures (S1 Text).

### Evaluation of clustering accuracy

For all pairs of genes featuring identical class labels, we assessed whether they belonged to the same cluster after clustering. The pairs of genes that featured identical class labels and belonged to the same cluster were true positives. The pairs of genes that featured distinct labels and belonged to different clusters were true negatives. The pairs of genes that featured the same labels but belonged to different clusters were false negatives. The pairs of genes that featured distinct labels but belonged to the same clusters were false positives. Thus, we converted the clustering problem into a binary classification problem. The true-positive rate (TPR; true positive / condition positive) and the false-positive rate (FPR; false positive / condition negative) were calculated at each merging step in hierarchical clustering. An ROC curve was created by plotting the TPR against the FPR, and the AUC of the ROC was used as a measure for clustering evaluation.

### Analysis for benchmark study of DEG detection

For the benchmark study of DEG detection, we used the same simulation datasets that were used for the benchmark study of clustering. In the same manner as the analysis for the benchmark study of clustering, the simulated reads were mapped to a mouse reference genome, the read counts of each gene per condition were obtained by using HTSeq, and edgeR, DESeq, DESeq2, and cuffdiff2 were applied for statistical testing for all pairs of conditions and to calculate the normalized expression values for each condition. Subsequently, DEclust was applied to divide 14,907 simulated genes into clusters by quitting the merge step if the distance between any pair of clusters *n*, *m* satisfied *D*(*C*_*n*_, *C*_*m*_) ≥ 1. Separately, the statistical tests for multiple conditions were performed by using edgeR, DESeq2, or multiDE [10] (version-1.0).

### Evaluation of DEG detection accuracy

For evaluation of the DEG detection power of DEclust, we defined a “DEG cluster” and “non-DEG cluster”. If at least one element of a pairwise DET profile of a cluster was 1 or −1, the cluster was a DEG cluster; otherwise (that is, all elements were 0), it was a non-DEG cluster. We defined whether a gene was selected as a DEG in a simulation as the correct label, and tested whether a gene belonged to a DEG cluster or non-DEG cluster obtained by using DEclust; therefore, if a gene was selected as a DEG and it belonged to any of the DEG clusters, it was a true positive. If a gene was not selected as a DEG and it belonged to any of the non-DEG clusters, it was a true negative. If a gene was not selected as a DEG but belonged to any of the DEG clusters, it was a false positive, and a gene selected as a DEG but that belonged to any of the non-DEG clusters was a false negative. For evaluation of the tools for detecting DEGs among multiple conditions, we defined whether a gene was selected as a DEG as the correct label, and tested whether a gene was detected as a DEG. Therefore, if a gene was simulated as a DEG and was detected as a DEG by a tool, it was true positive. If a gene was not simulated as a DEG and was not detected as a DEG, it was true negative. If a gene was not simulated as a DEG but was detected as a DEG, it was false-positive. If a gene was simulated as a DEG but was not detected as a DEG, it was false-negative. We evaluated edgeR, DESeq2, multiDE, and DEclust according to the true-positive rate (TPR), positive predictive rate (PPV), accuracy, and F-measure (harmonic mean of TPR and PPV).

### Mice

This study was conducted in strict accordance with the recommendations in the Guide for the Care and Use of Laboratory Animals of the Ministry of Education, Culture, Sports, Science, and Technology of Japan. The protocol was approved by the Committee on the Ethics of Animal Experiments of Chiba Cancer Center (Permit Number: 16–15). All efforts were made to minimize suffering. FVB/N mice were purchased from CLEA Japan, Tokyo, Japan.

### Skin carcinogenesis and tumor sampling

DMBA (7,12-dimethylbenz(a)anthracene) was used as a carcinogen and TPA (12-O-tetradecanoylphorbol-13-acetone) was used as a promoter. We treated 17 FVB/N mice according to a two-stage carcinogenesis protocol. At 8 weeks of age, the female mice were carefully shaved with electric clippers, and two days after shaving, a single dose of DMBA (25 μg/mouse in 200 μL of acetone) was applied to the shaved dorsal back skin. One week after initiation, tumors were promoted with TPA (10 μg/mouse in 200 μL of acetone) twice weekly for 20 weeks. The number and size (diameter, in mm) of each papilloma were recorded from 8 until 20 weeks, and carcinoma development was monitored for up to 40 weeks post-TPA treatment. The health of the mice was monitored every day by laboratory animal technicians. The mice were sacrificed by cervical dislocation when the tumor volume reaches 10% of the individual’s body weight. Lastly, normal skin, papilloma, carcinoma, and metastatic tumor samples were collected from each of two mice (two biological replicates, four stages), and the tumor volumes were calculated from the maximum diameter assuming a sphere (S12 Table).

### Sample preparation and mRNA-Seq

Total RNA was extracted from the papillomas, carcinomas, metastatic tumors, and normal tissues by using the AGPC (acid guanidinium thiocyanate-phenol-chloroform) method [20], and the total RNA samples were treated with DNase to eliminate genomic DNA. Each RNA sample was then reverse-transcribed using the SMARTER Ultra Low RNA Kit (634935; Takara, Otsu, Japan); 10 ng of total RNA was used and PCR was performed for 12 cycles. The products were treated according to the TruSeq DNA Sample Preparation v2 Guide for Illumina sequencing. In this step, PCR was performed for 15 cycles. Lastly, mRNA-Seq was performed using the Illumina Genome Analyzer IIx for eight samples (two replicates, four stages) according to the cBot workflow and Genome Analyzer IIx Paired End Run workflow. For each sample, 84 bp single-end reads were obtained.

### RNA-Seq data analysis

Low-quality mRNA-Seq reads were filtered by using FASTX-Toolkit (http://hannonlab.cshl.edu/fastx_toolkit/index.html) with the options -q 20 and -p 80. The remaining reads were mapped to the mouse reference genome (GRCm38-release71.fa) with gene annotation (Mus musculus.GRCm38.71.gtf) by using TopHat2 (version-2.0.8) with Bowtie2 (version-2.1.0). To identify significant differential expression, HTSeq (version-0.6.1) and DESeq2 (version-1.4.5) were used with the FDR controlled to <5%. DEclust was applied to the results of DESeq2, and a hierarchical tree of genes was calculated. The group average method with cosine distance was used for a secondary distance measure if any pairs of clusters featured the same distance under the definition of Eq (3) (S1 Text). The final clusters were determined by quitting the merge step if the distance between any pair of clusters *n*, *m* satisfied *D*(*C*_*n*_, *C*_*m*_) ≥ 1. The discriminative genes among carcinogenesis stages were investigated using a GSEA software [21] for each stage and cluster. Hallmark gene sets were used and the gene sets with an FDR q-value below 5% were identified; these were coherently expressed signatures derived by aggregating several Molecular Signatures Database gene sets. Moreover, the conventional hierarchical clustering method (the group average method with cosine distance) was applied to the detected DEGs for a comparison with the results obtained using DEclust. The hierarchical tree was cut into 16 clusters to obtain the same number of clusters as in the analysis with DEclust, and the GSEA was performed on each gene cluster.

## Supporting information

S1 Text Supporting information about DEclust algorithm and implementation. (DOCX)

S2 Text Entire description about simulation datasets. (DOCX)

S3 Text Description about evaluation of differential expression tests for constructing pairwise DET profiles. (DOCX)

S4 Text Discussion about accuracies of existing clustering methods in benchmark study. (DOCX)

S1 Fig Ten correct labels of benchmark study. Line plots with corresponding pairwise DET profiles. The handling of each dimension is same as Fig 1A. Four of the correct labels represent genes that are overexpressed in any one condition (a-d), while four other correct labels represent genes that are overexpressed in any two conditions (e-h). The rests of the labels represent genes that are overexpressed in any three conditions (i, j).
(TIF)

S2 Fig Result of clustering evaluation when using DESeq for statistical testing. *DEclust* is our method and *existing* are conventional hierarchical clustering methods. The vertical axis shows the mean AUC values, and the AUCs for each method for each number of replicates are plotted. The error bars are drawn in accordance with the corrected sample standard deviation of three simulations for each parameter set.
(TIF)

S3 Fig Result of clustering evaluation when using DESeq2 for statistical testing. *DEclust* is our method and *existing* are conventional hierarchical clustering methods. The vertical axis shows the mean AUC values, and the AUCs for each method for each number of replicates are plotted. The error bars are drawn in accordance with the corrected sample standard deviation of three simulations for each parameter set.
(TIF)

S4 Fig Result of clustering evaluation when using edgeR for statistical testing. *DEclust* is our method and *existing* are conventional hierarchical clustering methods. The vertical axis shows the mean AUC values, and the AUCs for each method for each number of replicates are plotted. The error bars are drawn in accordance with the corrected sample standard deviation of three simulations for each parameter set.
(TIF)

S5 Fig Result of clustering evaluation when using cuffdiff2 for statistical testing. *DEclust* is our method and *existing* are conventional hierarchical clustering methods. The vertical axis shows the mean AUC values, and the AUCs for each method for each number of replicates are plotted. The error bars are drawn in accordance with the corrected sample standard deviation of three simulations for each parameter set.
(TIF)

S6 Fig Evaluation results of differential expression test for constructing pairwise DET profile. The true-positive rate (TPR), false-positive rate (FPR), positive predictive value (PPV), and F-measure are plotted. The TPR, PPV, and F-measure are improved with an increase in the number of replicates.
(TIF)

S7 Fig Correlation between evaluation indicators of differential expression test and AUCs of clustering. Scatter-plot of the true-positive rate (TPR) against the AUC (a), scatter-plot of the positive predictive value (PPV) against the AUC (b), and scatter-plot of the F-measure against the AUC (c). These figures show that the TPR, PPV, and F-measure are highly correlated with AUC.
(TIF)

S8 Fig Results of DEG detection evaluation. The results for edgeR, DESeq2, multiDE, and DEclust are separately shown according to the F-measure, accuracy, true-positive rate, and positive predictive value. *DEclust*, our method, uses the statistical test results obtained from edgeR, DESeq, DESeq2, or cuffdiff2, and the evaluation results using each of these tools are separately shown as “DEclust_[DEGs detection tool]”.
(TIF)

S9 Fig Result of hierarchical clustering by using existing clustering method. (a) Hierarchical tree generated by the existing clustering method with the group average method for inter-cluster distance measure and the cosine distance for inter-gene distance measure. The clusters were divided at the highlighted in blue dotted lines. The cluster numbers are assigned to left to right cluster. (b) Line plots of the expression patterns for each gene in each cluster. The annotations in the bottom of figures are a cluster number associated with S11 Table and their pairwise DET profiles.
(TIF)

S1 Table Result of clustering evaluation when using DESeq for statistical testing. AUC values are shown in column 5–10.
(XLSX)

S2 Table Result of clustering evaluation when using DESeq2 for statistical testing. AUC values are shown in column 5–10.
(XLSX)

S3 Table Result of clustering evaluation when using edgeR for statistical testing. AUC values are shown in column 5–10.
(XLSX)

S4 Table Result of clustering evaluation when using cuffdiff2 for statistical testing. AUC values are shown in column 5–10.
(XLSX)

S5 Table Evaluation results of differential expression test for constructing pairwise DET profile. (XLSX)

S6 Table Results of DEG detection evaluation. (XLSX)

S7 Table Statistical summary of mouse mRNA-Seq data. An average of 40.10 M reads was obtained from mRNA-Seq with Illumina Genome Analyzer IIx. An average of 89.61% of reads passed a quality filter, and 82.81% of those reads were uniquely mapped to mouse reference genome.
(XLSX)

S8 Table Result of DEG detection for the mouse mRNA-Seq data. A total of 6,584 genes were detected as DEGs by DESeq2.
(XLSX)

S9 Table Details of the 16 clusters obtained by using DEclust for mouse transcriptome data. (XLSX)

S10 Table Result of GSEA for each cluster obtained by using DEclust. The column “# genes” represents the number of genes for each cluster. In this table, the ‘N’ means normal skin, ‘P’ means papilloma, ‘C’ means carcinoma, and ‘M’ means metastatic tumor. The six-dimensional patterns are corresponding to the pairwise DET profiles for each cluster. The first dimension, ‘NP’, denotes the statistical profile between normal skin and papilloma in certain cluster. The reference condition is whichever character comes first, so the first dimension is 1 if the expression level in papilloma is significantly higher than that in normal skin. The column “Explanation” represents an interpretation of the pairwise DET profile, and the column “GSEA Hallmarks” shows the top three hallmarks for each cluster.
(XLSX)

S11 Table Result of GSEA for each cluster obtained using existing method. In the same manner as S10 Table, the ‘N’, ‘P’, ‘C’, and ‘M’ are corresponding to the normal skin, papilloma, carcinoma, and metastatic tumor, relatively.
(XLSX)

S12 Table Volumes of tumor samples. (XLSX)

S1 Algorithm Pseudo code of DEclust. (DOCX)
