# Supplementary material for: DEclust: A statistical approach for obtaining differential expression profiles of multiple conditions
Source: PLoS One. 2017 Nov 21;12(11):e0188285. doi: 10.1371/journal.pone.0188285 (PMC5697878; doi:10.1371/journal.pone.0188285)
Supplement: S1 Text — (DOCX) [file pone.0188285.s001.docx]

**S1 Text. Supporting information about DEclust algorithm and implementation.**

We define a collection of statistical test results for differential expression obtained for all combinatorial pairs of conditions as *pairwise differential expression test (DET) profile* and assign it to each gene (equation (1)). The pairwise DET profile is extended to a set (cluster) of genes (equation (2)), and used by DEclust for searching for a set of genes whose expression profiles (pairwise DET profiles) are statistically overrepresented among multiple conditions. DEclust adopts the agglomerative-hierarchical-type clustering method based on the definition of distance between gene clusters (equation (3)). If any pairs of clusters have the same distance under the definition of equation (3), then DEclust uses a conventional distance measure to search for the closest pair of clusters. As a conventional inter-cluster distance measure, the group average method, single-linkage method, complete-linkage method, and Ward’s method [1] are implemented in DEclust. As a conventional inter-gene distance measure, Euclidean distance, Pearson’s correlation, and cosine distance are implemented in DEclust.

**Reference**

1. Ward JH. Hierarchical grouping to optimize an objective function. Journal of the American Statistical Association. 1963. pp. 236–244. doi:10.1080/01621459.1963.10500845
