# Supplementary material for: DEclust: A statistical approach for obtaining differential expression profiles of multiple conditions
Source: PLoS One. 2017 Nov 21;12(11):e0188285. doi: 10.1371/journal.pone.0188285 (PMC5697878; doi:10.1371/journal.pone.0188285)
Supplement: S2 Text — (DOCX) [file pone.0188285.s002.docx]

**S2 Text. Entire description about simulation datasets.**

To evaluate DEclust and conventional clustering methods, we generated simulated short reads from mouse reference cDNA sequences so that gene expression profiles for four artificial conditions were obtained. We set up ten pairwise DET profiles as correct labels for DEGs (S1 Fig). Four of the correct labels represent genes that are overexpressed in any one condition (clusters 1-4), while four other correct labels represent genes that are overexpressed in any two conditions (clusters 5-8). The rests of the correct labels represent genes that are overexpressed in any three conditions (clusters 9 and 10). Second, 20% of genes were randomly selected as DEGs and the correct label for DEGs was randomly assigned for each selected gene. Other genes were classified in cluster 0 whose pairwise DET profile is defined as (0 0 0 0 0 0), indicating that the expression levels of any pairs of conditions are not significantly different. Third, a control distribution that follows the negative binomial distribution was generated for each gene. The RNA-Seq read counts for each condition were sampled as many times as the number of replicates from the control distribution, and the read counts for differentially expressed conditions were biased by a randomly selected fold-change. The fold-change was selected from a pool of $(\times1.5, \times2.0, \times2.5, \times3.0)$.

We generated the control distribution for each gene as follows. First, we downloaded a mouse reference genome, mouse reference cDNA, and mouse gene annotation file from Ensembl (http://www.ensembl.org/index. html, GRCm38-release71), and RNA-Seq read count data of 69 Nigerian lymphoblastoid cell lines (LCLs) from Pickrell et al. [1] to yield practical datasets. We extracted genes whose total exon lengths are greater than 200bp from the Pickrell et al. data. We calculated a fragments per kilobase of exons per million mapped fragments (FPKM) [2] for each gene as follows:

| $\mathrm{FPKM}_{i,j}=\frac{{10}^{9}x_{i,j}}{M_{j}L_{i}},$ | (S1) |
| --- | --- |

The FPKM is commonly used to convert the RNA-Seq read counts to comparable normalized expression values. Where $x_{i,j}$ represents the number of reads mapped to a gene $i$ of a sample $j$. $M_{j}$ is a total number of reads for a sample $j$, and $L_{i}$ is a total exon length of a gene $i$. From the FPKM of each sample, the mean and variance of the FPKM values for each gene were calculated and paired as FPKM statistics data. The FPKM statistics data were filtered if the means of the FPKM were less than one. Second, the FPKM statistics data and mouse cDNA sequence were matched so that the total exon length of the original gene of the FPKM statistics data and the total length of the mouse cDNA sequence are nearest. As a result, 14,907 combinations of the FPKM statistics data and mouse cDNA sequence were obtained.

The control distributions were generated based on the FPKM statistics data, which contain the mean and variance of the FPKM values. We calculated the mean and variance of read counts from the mean and variance of FPKM values as follows (the detail of an equation conversion is shown at the end of this section):

| $E\left[ x_{i,j\in k} \right]=\frac{M_{k}L_{i}}{{10}^{9}}E\left[ \mathrm{FPKM}_{i,j\in k} \right]$ | (S2) |
| --- | --- |
| $Var\left[ x_{i,j\in k} \right]=\left( \frac{M_{k}L_{i}}{{10}^{9}} \right)^{2}Var\left[ \mathrm{FPKM}_{i,j\in k} \right],$ | (S3) |

where $M_{k}$ represents the number of total reads for any samples of a condition $k$. In this benchmark study, the numbers of the total reads were assumed to be evenly distributed among samples. According to the mean and variance of the read counts, we generated a probabilistic distribution as the control distribution, which follows the negative binomial distribution. Finally, the read counts were sampled from the control distribution as many times as the number of replicates, and the RNA-Seq reads were generated from the matched mouse cDNA sequence as mentioned above. Thus, we simulated 14,907 mouse transcripts (genes) RNA-Seq datasets for our benchmark study.

As to generate datasets, the total number of reads for each sample was simulated approximately $1.0\times{10}^{7}$, the single-end RNA-Seq reads with 120 bp of length were generated in accordance with the read counts. For each condition, the sampling of the read count was repeated with the number of replicates progressively ranging from 1-6, 12, 18, and 24. Moreover, we simulated three times for each parameter set.

At the end of this section, the equation conversion from the mean and variance of the FPKM (equation S1) to the mean and variance of read counts can be performed as follows:

| $E\left[ \mathrm{FPKM}_{i,j\in k} \right]=\frac{1}{N}\sum_{a\in k} \left( \frac{{10}^{9}x_{i,a}}{M_{k}L_{i}} \right)$ $=\frac{{10}^{9}}{M_{k}L_{i}}\frac{1}{N}\sum_{a\in k} x_{i,a}$ $=\frac{{10}^{9}}{M_{k}L_{i}}E\left[ x_{i,j\in k} \right]$ $\therefore E\left[ x_{i,j\in k} \right]=\frac{M_{k}L_{i}}{{10}^{9}}E\left[ \mathrm{FPKM}_{i,j\in k} \right]$ |  |
| --- | --- |
| $Var\left[ \mathrm{FPKM}_{i,j\in k} \right]=\frac{1}{N-1}\sum_{a\in k} \left( E\left[ \mathrm{FPKM}_{i,j\in k} \right]-\mathrm{FPK}M_{i,a} \right)^{2}$ $=\frac{\left( {{10}^{9}}/{M_{k}L_{i}} \right)^{2}}{N-1}\sum_{a\in k} \left( \frac{1}{N}\sum_{l\in k} x_{i,l}-x_{i,a} \right)^{2}$ $=\left( {{10}^{9}}/{M_{k}L_{i}} \right)^{2} Var\left[ x_{i,j\in k} \right]$  $\therefore Var\left[ x_{i,j\in k} \right]=\left( \frac{M_{k}L_{i}}{{10}^{9}} \right)^{2}Var\left[ \mathrm{FPKM}_{i,j\in k} \right]$ |  |

Reference

1. Pickrell J, Marioni J, Pai A, Degner JF, Engelhardt BE. Understanding mechanisms underlying human gene expression variation with RNA sequencing. Nature. 2010;464: 768–772. doi:10.1038/nature08872.Understanding

2. Trapnell C, Williams B a, Pertea G, Mortazavi A, Kwan G, van Baren MJ, et al. Transcript assembly and quantification by RNA-Seq reveals unannotated transcripts and isoform switching during cell differentiation. Nat Biotechnol. 2010;28: 511–515. doi:10.1038/nbt.1621
