# Supplementary material for: DEclust: A statistical approach for obtaining differential expression profiles of multiple conditions
Source: PLoS One. 2017 Nov 21;12(11):e0188285. doi: 10.1371/journal.pone.0188285 (PMC5697878; doi:10.1371/journal.pone.0188285)
Supplement: S3 Text — (DOCX) [file pone.0188285.s003.docx]

**S3 Text. Description about evaluation of differential expression tests for constructing pairwise DET profiles.**

We evaluated the statistical test results of edgeR [1], DESeq [2], DESeq2 [3], and cuffdiff2 [4], because DEclust crucially depends on the statistical test results. We assessed whether a pairwise DET profile composed of all pairwise statistical test results from differential expression analysis tools is exactly matched with the correct pairwise DET profile (correct class label) for each gene. If these pairwise DET profiles matched exactly, they were true; otherwise, they were false. For a certain condition *k*, *l*, and a certain gene *g*, if the $d_{k,l}^{g}$ of the correct class label (pairwise DET profile) was 0 and the statistical test outcome of the differential expression analysis tool was significant (it means that the expression levels of gene *g* was significantly different between the condition *k* and *l*), it is false positive. In contrast, if the $d_{k,l}^{g}$ of the correct class label was 1 or -1 and the statistical test outcome was insignificant; it is false negative. We calculated the true positive rate (TPR; true positive / condition positive), false positive rate (FPR; false positive / condition negative), positive predictive value (PPV; true positive / test outcome positive), and F-measure, which is a harmonic mean of the TPR and PPV. Whichever statistical analysis tool was used, the TPR, PPV, and F-measure were improved with the increase in the number of replicates (S6 Fig, and S5 Table). The TPR, PPV, and F-measure were highly correlated with the AUC of DEclust (S7 Fig). This implies that the increase of the number of replicates affects the improvement of statistical test accuracy, and so the cluster analysis using DEclust achieves high accuracy with more replicates.

Reference

1. Robinson MD, McCarthy DJ, Smyth GK. edgeR: a Bioconductor package for differential expression analysis of digital gene expression data. Bioinformatics. 2010;26: 139–140. doi:10.1093/bioinformatics/btp616

2. Anders S, Huber W. Differential expression analysis for sequence count data. Genome Biol. 2010;11: R106. doi:10.1186/gb-2010-11-10-r106

3. Love MI, Huber W, Anders S. Moderated estimation of fold change and dispersion for RNA-seq data with DESeq2. Genome Biol. 2014;15: 550. doi:10.1186/s13059-014-0550-8

4. Trapnell C, Hendrickson DG, Sauvageau M, Goff L, Rinn JL, Pachter L. Differential analysis of gene regulation at transcript resolution with RNA-seq. Nat Biotechnol. 2013;31: 46–53. doi:10.1038/nbt.2450
