# Supplementary material for: DEclust: A statistical approach for obtaining differential expression profiles of multiple conditions
Source: PLoS One. 2017 Nov 21;12(11):e0188285. doi: 10.1371/journal.pone.0188285 (PMC5697878; doi:10.1371/journal.pone.0188285)
Supplement: S4 Text — (DOCX) [file pone.0188285.s004.docx]

**S4 Text: Discussion about accuracies of existing clustering methods in benchmark study.**

Fig 2, S2-S5 Figs shows that the accuracy almost never changed if Euclidean distance or Pearson’s correlation was used for the inter-gene distance measure, but the accuracy was improved with an increase in the number of replicates when the cosine distance was used for the inter-gene distance measure. Initially, we presumed that the estimation accuracy of gene expression levels became more precise in accordance with the number of replicates, so the classification accuracy should be progressively improved with increasing the number of replicates, although, the accuracy of the existing methods using Euclidean distance and Pearson’s correlation almost never changed at a lower level.

Fundamentally, Euclidean distance does not consider the correlation between gene expression patterns, so it could not cluster genes whose increasing and decreasing patterns of the expression level among multiple conditions were similar. On the other hand, cosine distance considers an inner product between the gene expression patterns so that it assesses the correlation between expression patterns. The Pearson’s correlation is also a normalized cosine similarity. The normalization is performed by subtracting the mean of expression levels under the assumption that those expression levels are derived from the normal distribution; however, it is known that the normal distribution is not suitable for modeling the gene expression level [1]. Moreover, the expression levels of DEGs can be considered as a multimodal distribution as they are significantly different between conditions; the assumption about the distribution of gene expression is partially reliable. Thus, we assume that the accuracy of the existing clustering method using Pearson’s correlation would not advance with the increase in the number of replicates.

Reference

1. Trapnell C, Williams B a, Pertea G, Mortazavi A, Kwan G, van Baren MJ, et al. Transcript assembly and quantification by RNA-Seq reveals unannotated transcripts and isoform switching during cell differentiation. Nat Biotechnol. 2010;28: 511–515. doi:10.1038/nbt.1621
