# Supplementary figures and images for: DEclust: A statistical approach for obtaining differential expression profiles of multiple conditions
Source: PLoS One. 2017 Nov 21;12(11):e0188285. doi: 10.1371/journal.pone.0188285 (PMC5697878; doi:10.1371/journal.pone.0188285)

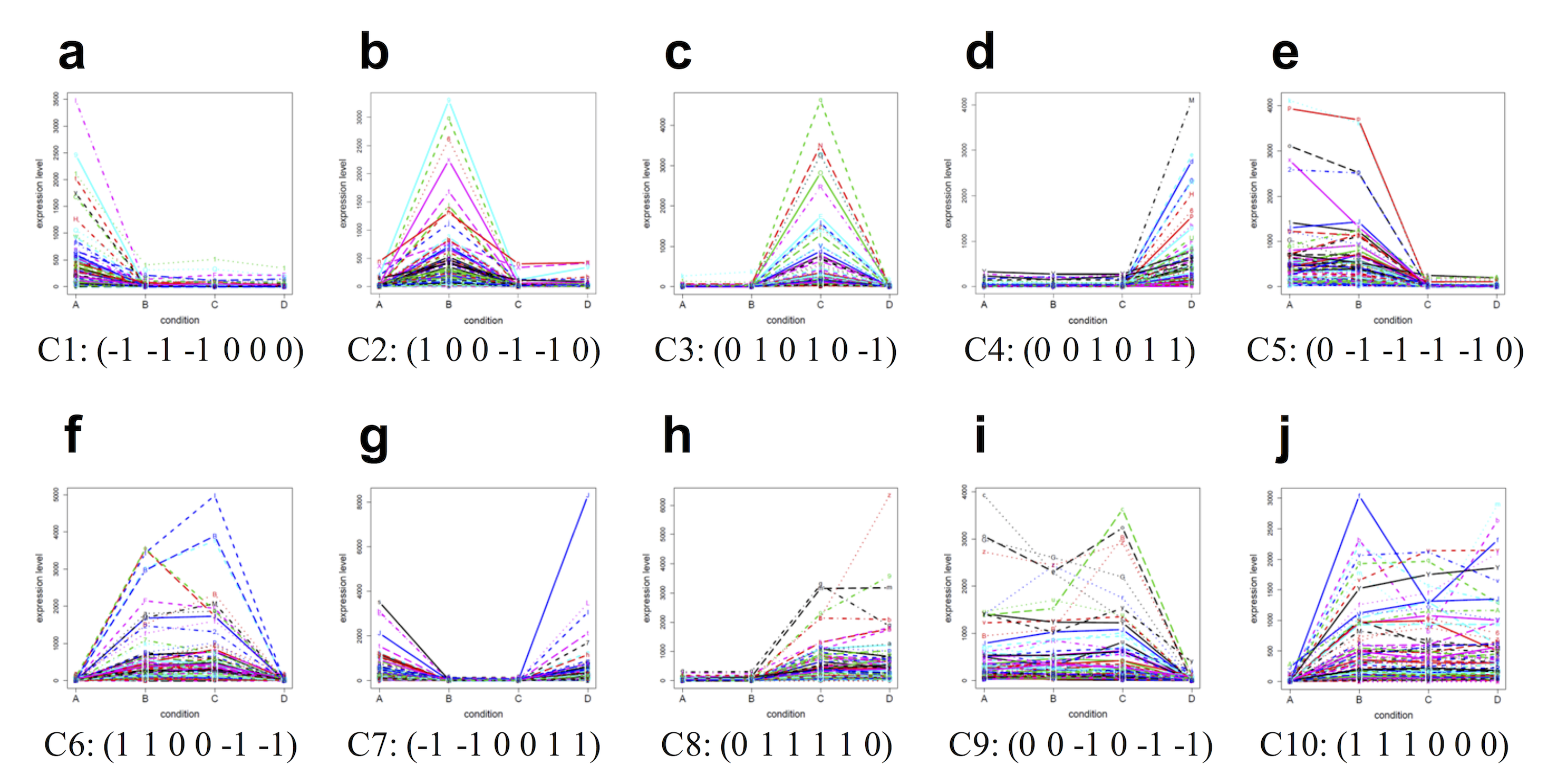

Supplement: S1 Fig — Line plots with corresponding pairwise DET profiles. The handling of each dimension is same as Fig 1A. Four of the correct labels represent genes that are overexpressed in any one condition (a-d), while four other correct labels represent genes that are overexpressed in any two conditions (e-h). The rests of the labels represent genes that are overexpressed in any three conditions (i, j). (TIF) [file pone.0188285.s005.tif]

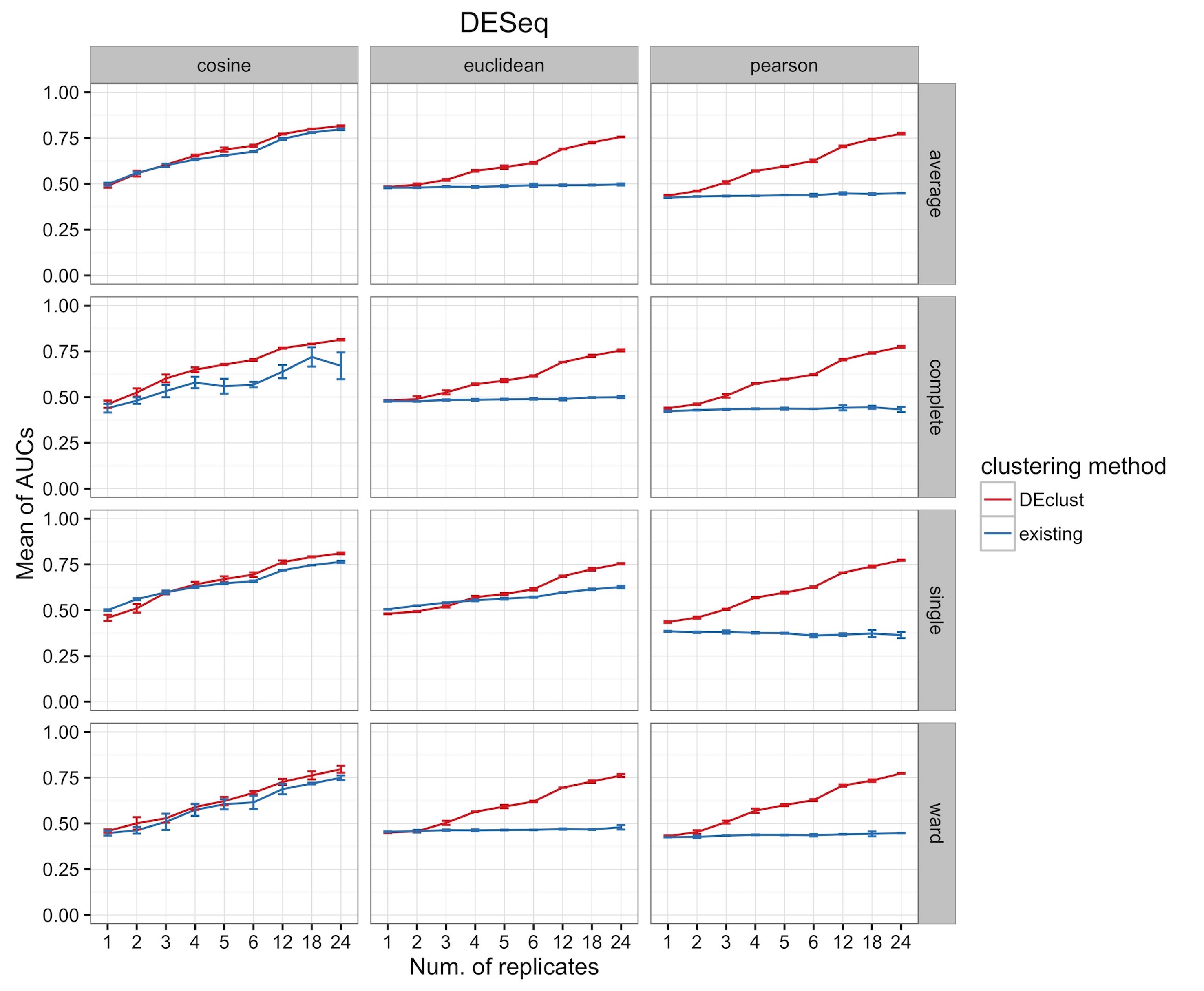

Supplement: S2 Fig — DEclust is our method and existing are conventional hierarchical clustering methods. The vertical axis shows the mean AUC values, and the AUCs for each method for each number of replicates are plotted. The error bars are drawn in accordance with the corrected sample standard deviation of three simulations for each parameter set. (TIF) [file pone.0188285.s006.tif]

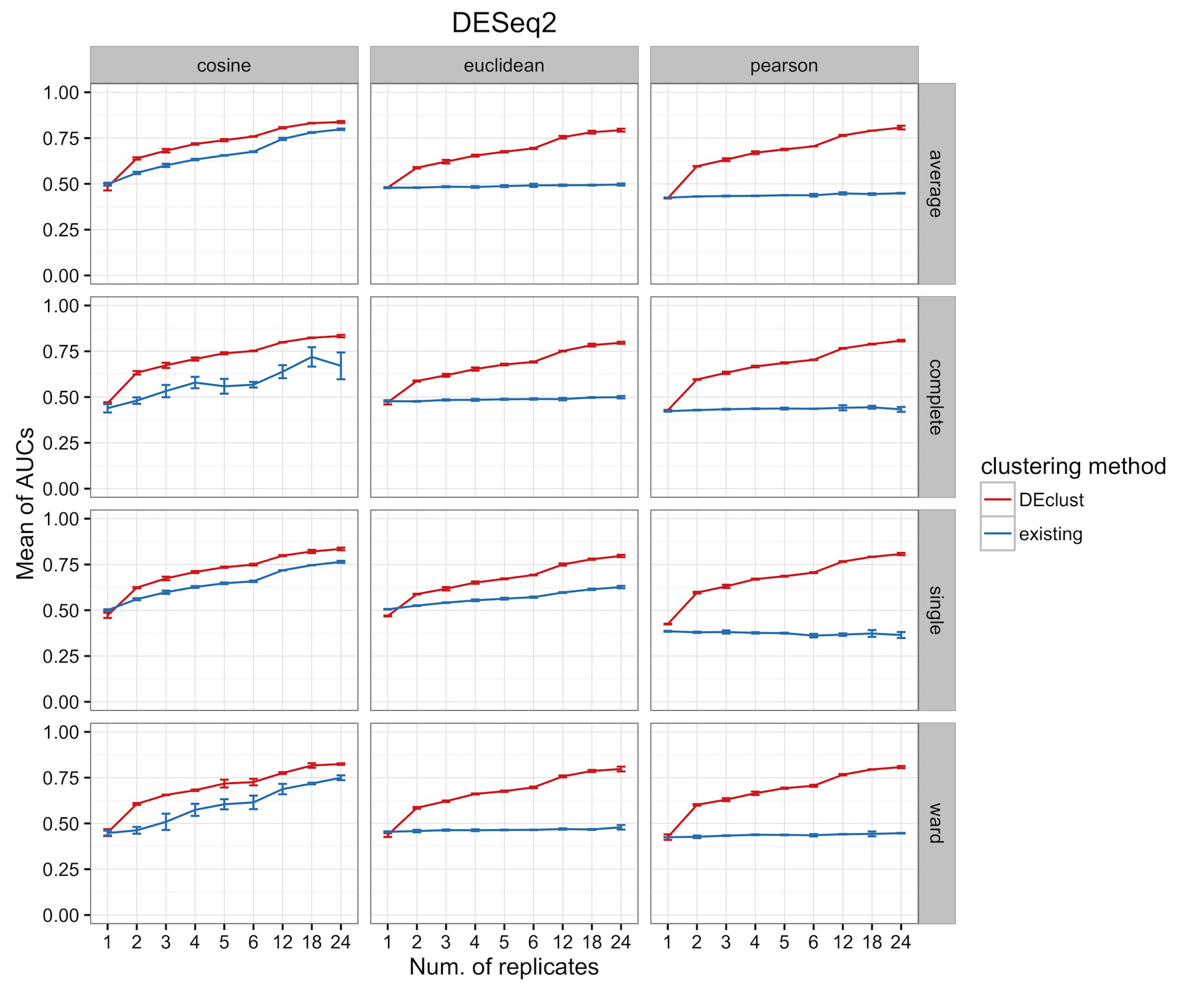

Supplement: S3 Fig — DEclust is our method and existing are conventional hierarchical clustering methods. The vertical axis shows the mean AUC values, and the AUCs for each method for each number of replicates are plotted. The error bars are drawn in accordance with the corrected sample standard deviation of three simulations for each parameter set. (TIF) [file pone.0188285.s007.tif]

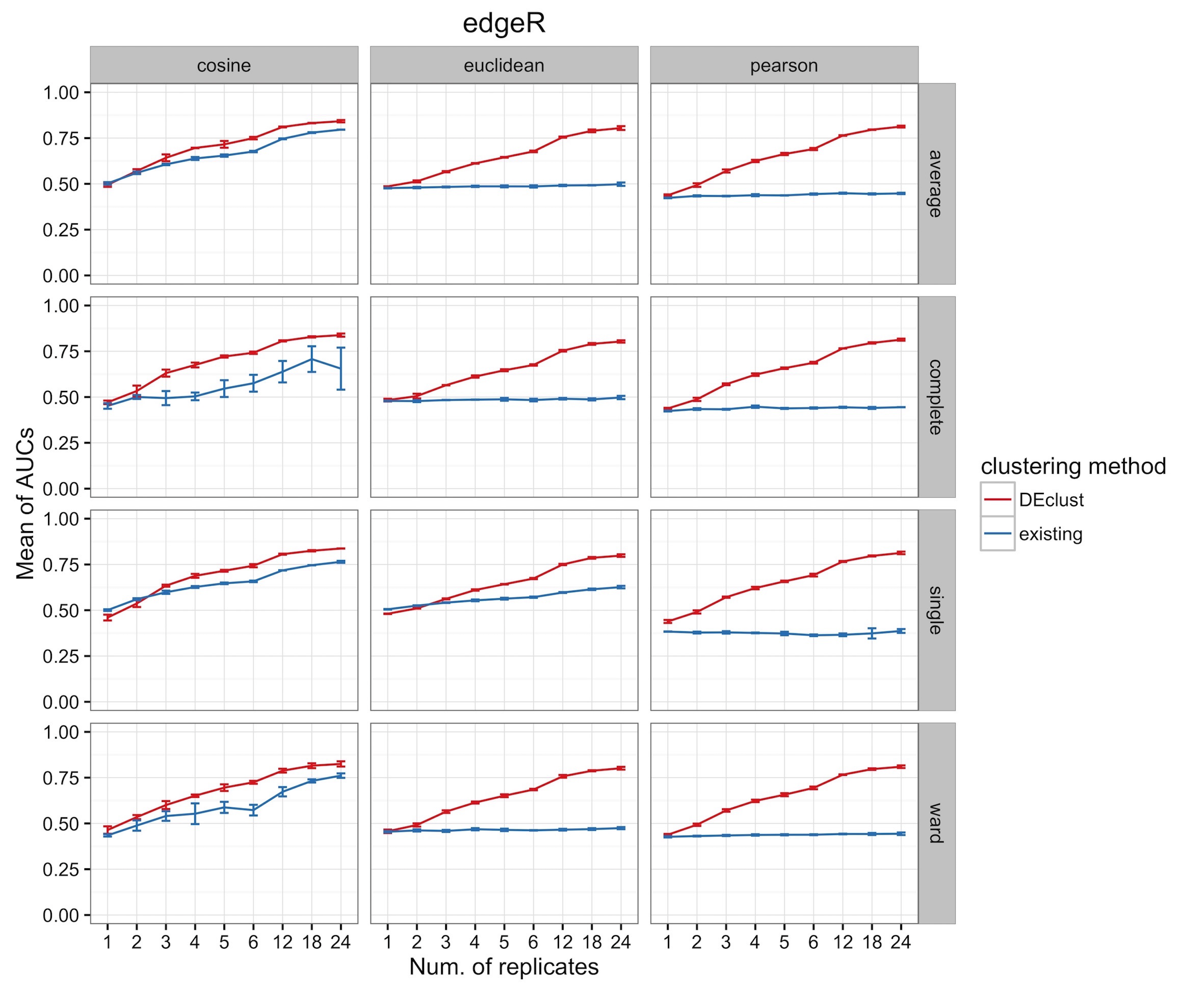

Supplement: S4 Fig — DEclust is our method and existing are conventional hierarchical clustering methods. The vertical axis shows the mean AUC values, and the AUCs for each method for each number of replicates are plotted. The error bars are drawn in accordance with the corrected sample standard deviation of three simulations for each parameter set. (TIF) [file pone.0188285.s008.tif]

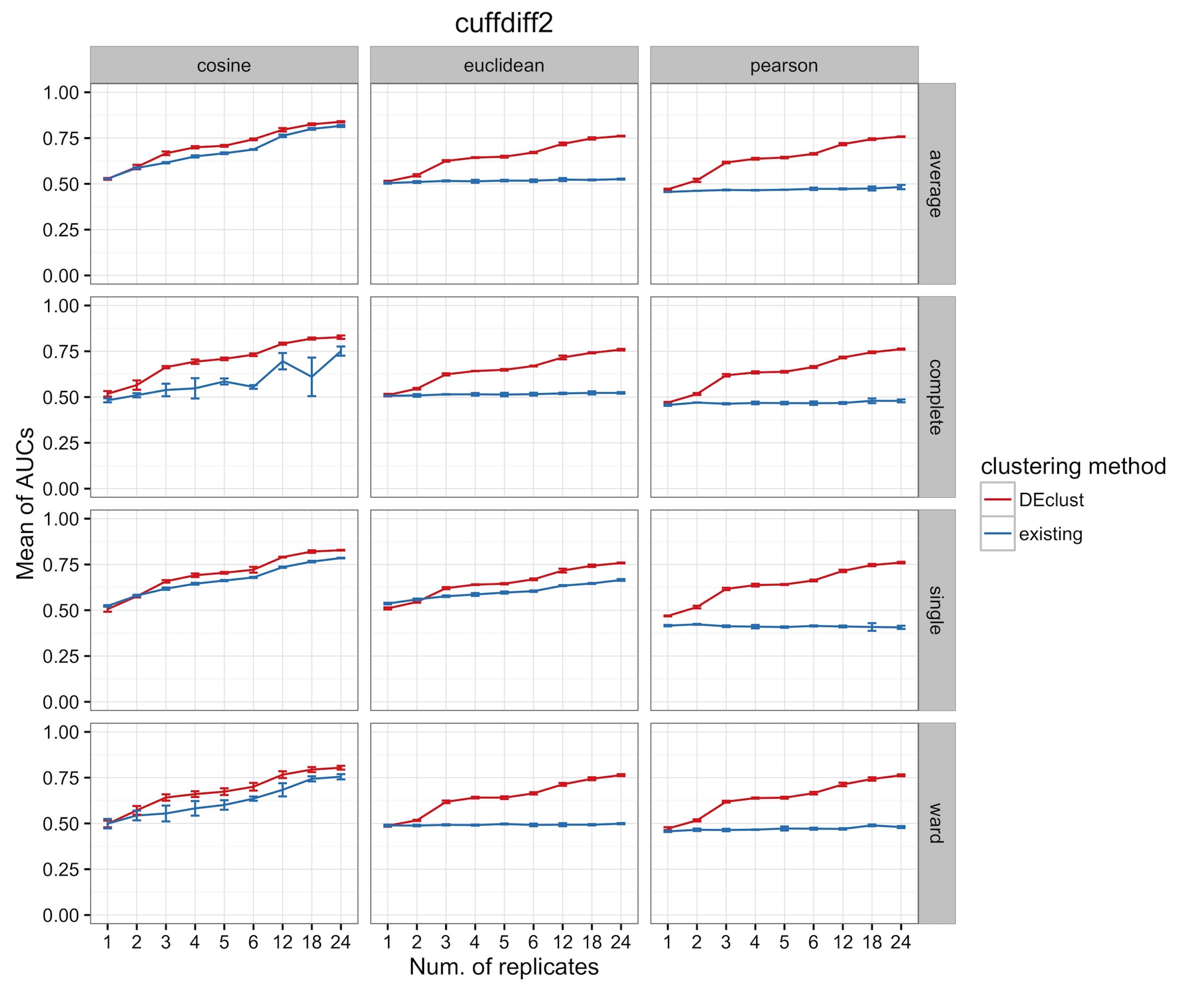

Supplement: S5 Fig — DEclust is our method and existing are conventional hierarchical clustering methods. The vertical axis shows the mean AUC values, and the AUCs for each method for each number of replicates are plotted. The error bars are drawn in accordance with the corrected sample standard deviation of three simulations for each parameter set. (TIF) [file pone.0188285.s009.tif]

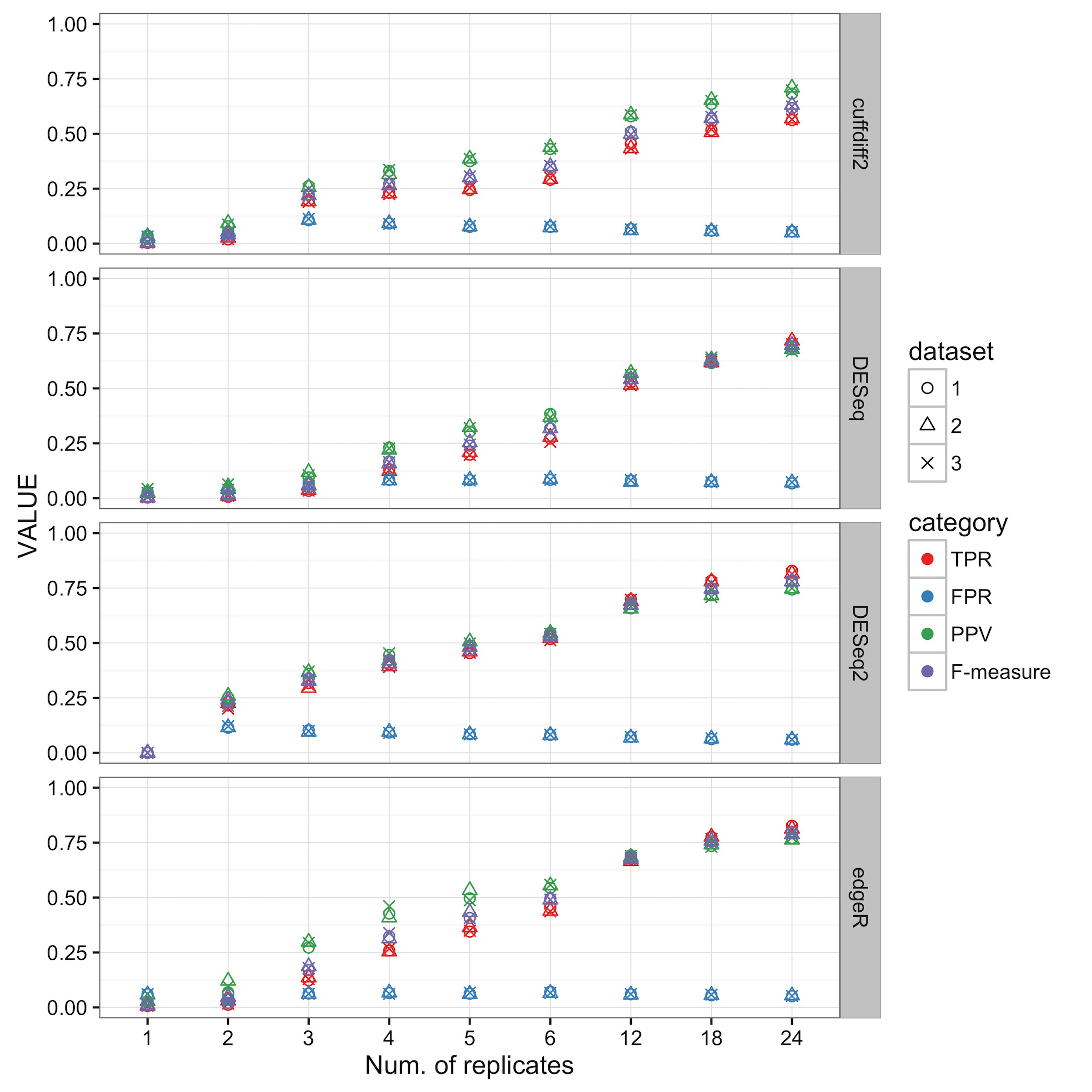

Supplement: S6 Fig — The true-positive rate (TPR), false-positive rate (FPR), positive predictive value (PPV), and F-measure are plotted. The TPR, PPV, and F-measure are improved with an increase in the number of replicates. (TIF) [file pone.0188285.s010.tif]

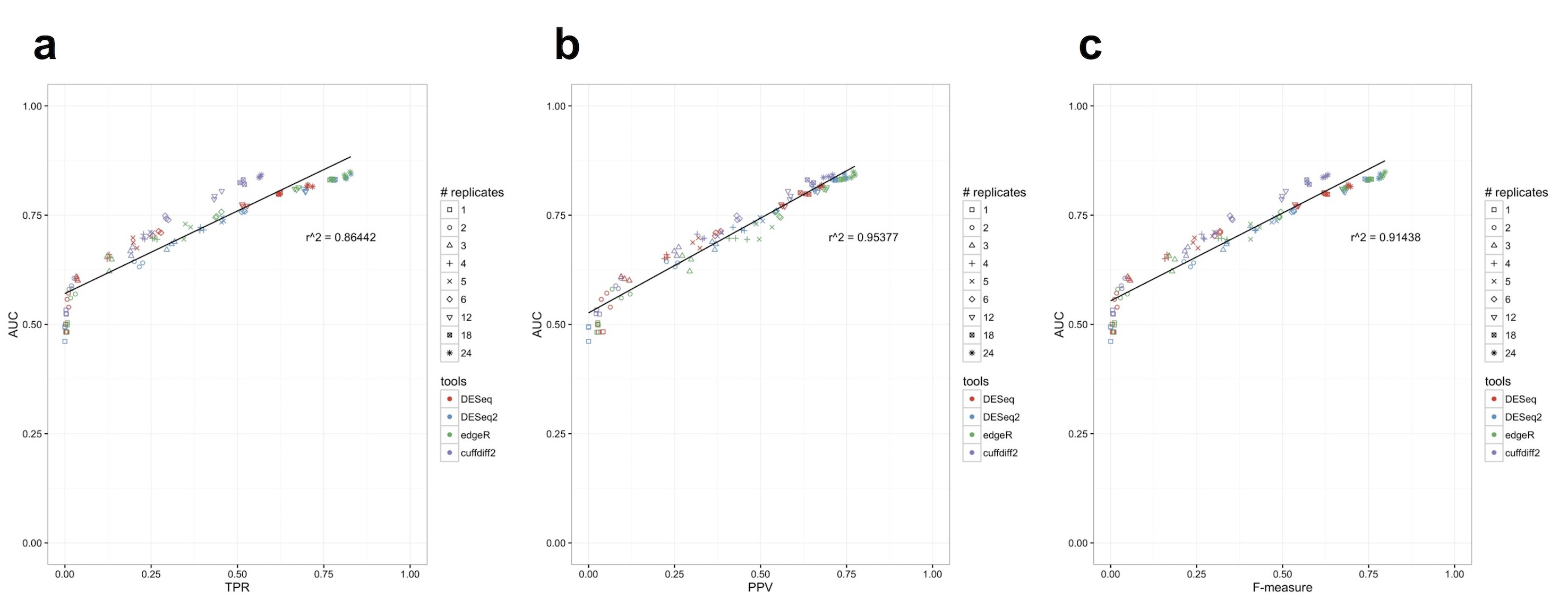

Supplement: S7 Fig — Scatter-plot of the true-positive rate (TPR) against the AUC (a), scatter-plot of the positive predictive value (PPV) against the AUC (b), and scatter-plot of the F-measure against the AUC (c). These figures show that the TPR, PPV, and F-measure are highly correlated with AUC. (TIF) [file pone.0188285.s011.tif]

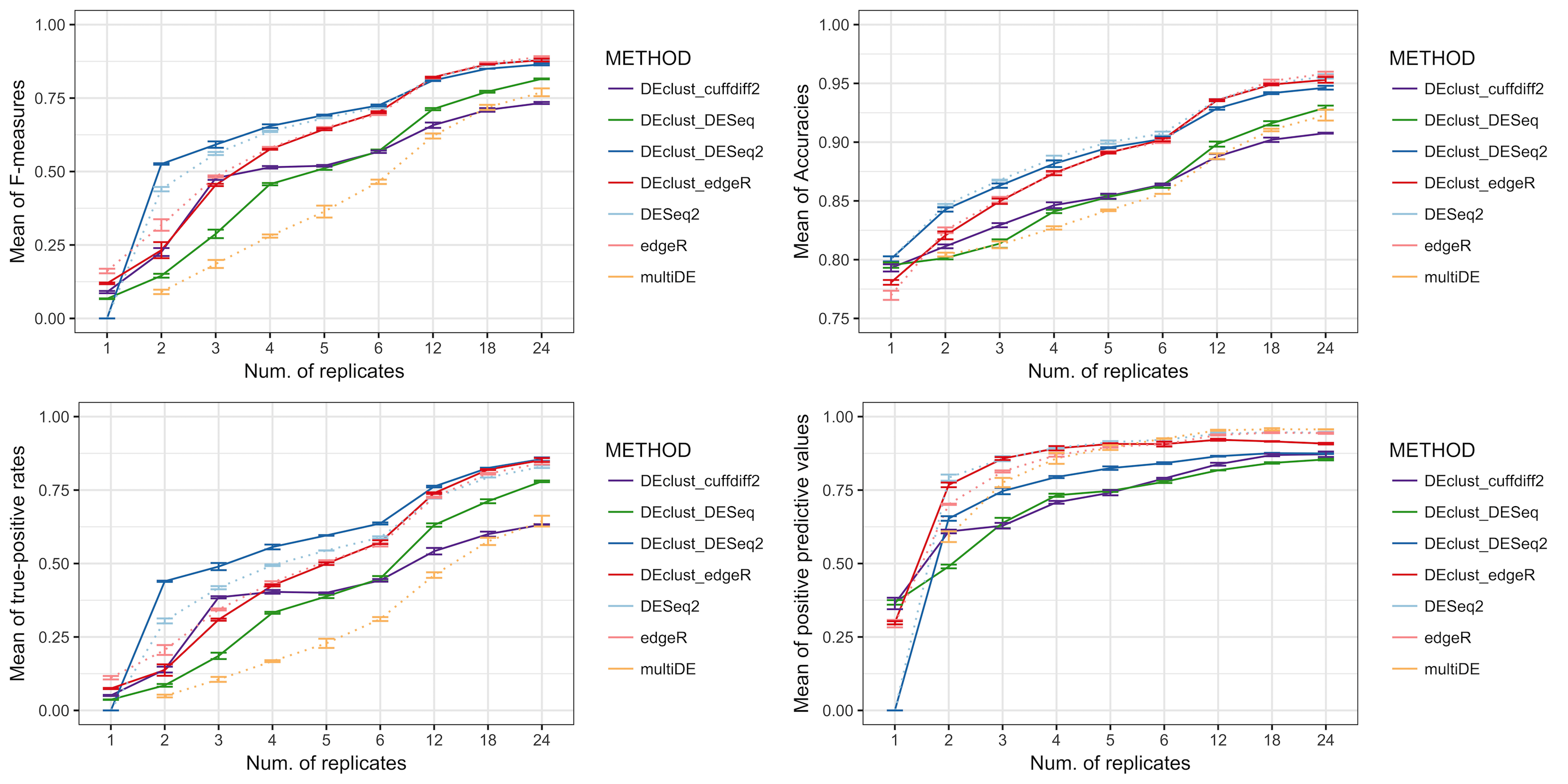

Supplement: S8 Fig — The results for edgeR, DESeq2, multiDE, and DEclust are separately shown according to the F-measure, accuracy, true-positive rate, and positive predictive value. DEclust, our method, uses the statistical test results obtained from edgeR, DESeq, DESeq2, or cuffdiff2, and the evaluation results using each of these tools are separately shown as “DEclust_[DEGs detection tool]”. (TIF) [file pone.0188285.s012.tif]

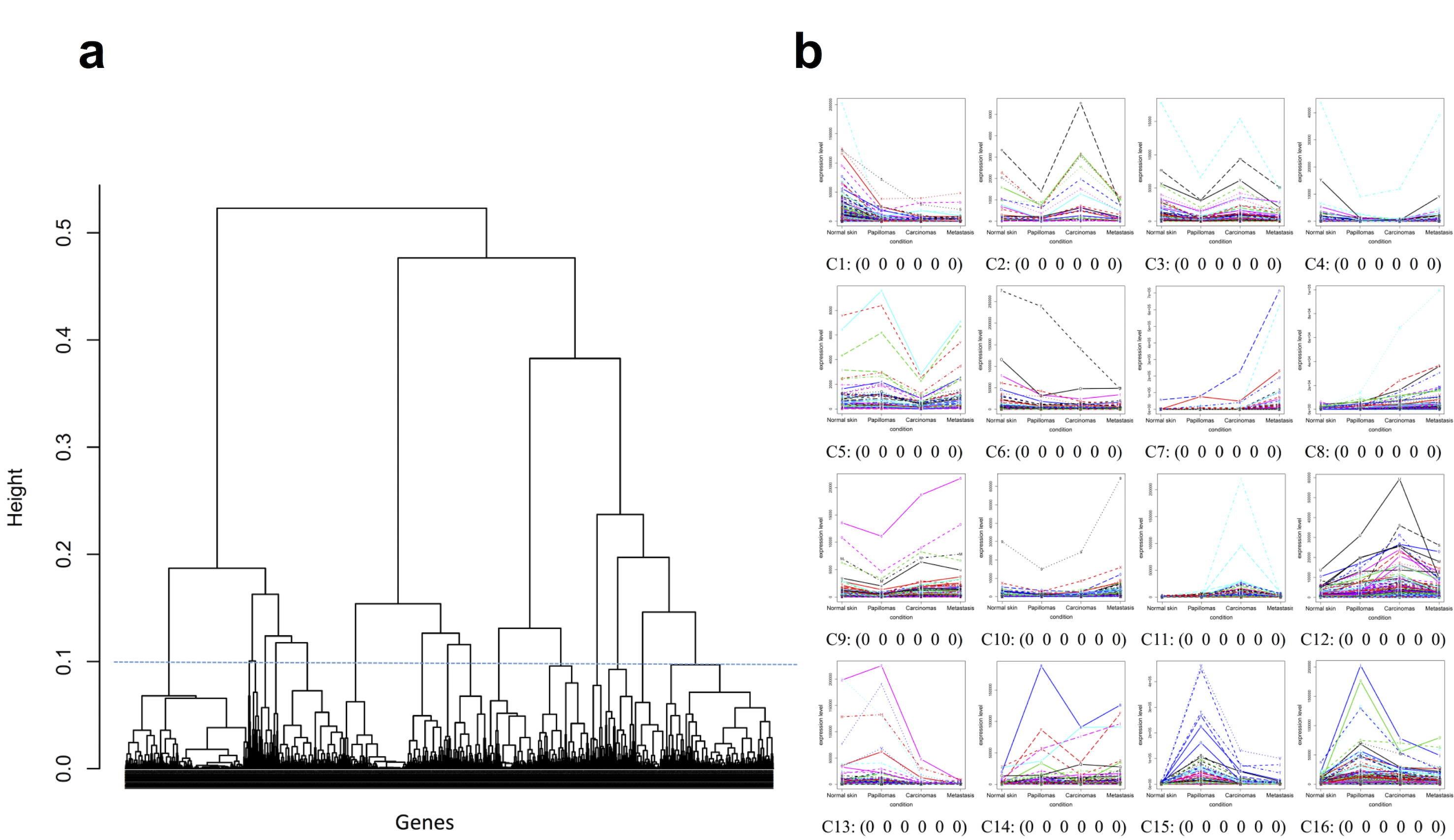

Supplement: S9 Fig — (a) Hierarchical tree generated by the existing clustering method with the group average method for inter-cluster distance measure and the cosine distance for inter-gene distance measure. The clusters were divided at the highlighted in blue dotted lines. The cluster numbers are assigned to left to right cluster. (b) Line plots of the expression patterns for each gene in each cluster. The annotations in the bottom of figures are a cluster number associated with S11 Table and their pairwise DET profiles. (TIF) [file pone.0188285.s013.tif]
