# Supplementary material for: DEclust: A statistical approach for obtaining differential expression profiles of multiple conditions
Source: PLoS One. 2017 Nov 21;12(11):e0188285. doi: 10.1371/journal.pone.0188285 (PMC5697878; doi:10.1371/journal.pone.0188285)
Supplement: S1 Algorithm — (DOCX) [file pone.0188285.s026.docx]

| **S1 Algorithm. Pseudo code of DEclust.** |
| --- |
| **Input**: $G=\{g_{1}, g_{2}, \ldots, g_{n}\}$ (set of genes to be clustered). |
| **Output**: *N* (Hierarchical tree written in Newick tree format). |
| **Procedure**:   Assign one gene to each cluster.  Calculate the distance *D* according to equation (3) for all pairs of clusters. |
| **while** there is more than one cluster **do**  Search for a pair of clusters with the minimum distance.  **If** Any pairs of clusters have the same distance **then**  Compare conventional distance of expression levels.  **end if**  Merge the clusters.  Store the clusters and the distance *D* in Newick format to *N*.  Calculate the distance *D* using the equation (3) between a new cluster and other clusters.  **end while**  **return** *N* |
